# Chronic Nicotine Exposure Induces Murine Aortic Remodeling and Stiffness Segmentation—Implications for Abdominal Aortic Aneurysm Susceptibility

**DOI:** 10.3389/fphys.2018.01459

**Published:** 2018-10-31

**Authors:** Markus U. Wagenhäuser, Isabel N. Schellinger, Takuya Yoshino, Kensuke Toyama, Yosuke Kayama, Alicia Deng, Sabina P. Guenther, Anne Petzold, Joscha Mulorz, Pireyatharsheny Mulorz, Gerd Hasenfuß, Wiebke Ibing, Margitta Elvers, Andreas Schuster, Anand K. Ramasubramanian, Matti Adam, Hubert Schelzig, Joshua M. Spin, Uwe Raaz, Philip S. Tsao

**Affiliations:** ^1^Cardiovascular Institute, Stanford University School of Medicine, Stanford, CA, United States; ^2^VA Palo Alto Health Care System, Palo Alto, CA, United States; ^3^Department of Vascular and Endovascular Surgery, University Hospital Düsseldorf, Heinrich-Heine-University, Düsseldorf, Germany; ^4^Molecular and Translational Vascular Medicine, Department of Cardiology and Pneumology, Heart Center at the University Medical Center Göttingen, Göttingen, Germany; ^5^German Center for Cardiovascular Research e.V., Göttingen, Germany; ^6^Department of Endocrinology and Nephrology, University of Leipzig, Leipzig, Germany; ^7^Department of Cardiac Surgery, University Hospital Munich, Ludwig-Maximilian-University, Munich, Germany; ^8^Department of Surgery, University of California, San Francisco, San Francisco, CA, United States; ^9^Department of Cardiology, Royal North Shore Hospital, The Kolling Institute, Northern Clinical School, University of Sydney, Sydney, NSW, Australia; ^10^Department of Biomedical, Chemical and Materials Engineering, San Jose State University, San Jose, CA, United States

**Keywords:** nicotine, AAA, stiffness, segmentation, e-cigarettes, mouse model

## Abstract

**Aim:** Arterial stiffness is a significant risk factor for many cardiovascular diseases, including abdominal aortic aneurysms (AAA). Nicotine, the major active ingredient of e-cigarettes and tobacco smoke, induces acute vasomotor effects that may temporarily increase arterial stiffness. Here, we investigated the effects of long-term nicotine exposure on structural aortic stiffness.

**Methods:** Mice (C57BL/6) were infused with nicotine for 40 days (20 mg/kg/day). Arterial stiffness of the thoracic (TS) and abdominal (AS) aortic segments was analyzed using ultrasound (PWV, pulse wave velocity) and *ex vivo* pressure myograph measurements. For mechanistic studies, aortic matrix-metalloproteinase (MMP) expression and activity as well as medial elastin architecture were analyzed.

**Results:** Global aortic stiffness increased with nicotine. In particular, local stiffening of the abdominal segment occurred after 10 days, while thoracic aortic stiffness was only increased after 40 days, resulting in aortic stiffness segmentation. Mechanistically, nicotine exposure enhanced expression of MMP-2/-9 and elastolytic activity in both aortic segments. Elastin degradation occurred in both segments; however, basal elastin levels were higher in the thoracic aorta. Finally, MMP-inhibition significantly reduced nicotine-induced MMP activity, elastin destruction, and aortic stiffening.

**Conclusion:** Chronic nicotine exposure induces aortic MMP expression and structural aortic damage (elastin fragmentation), irreversibly increasing aortic stiffness. This process predominantly affects the abdominal aortic segment, presumably due in part to a lower basal elastin content. This novel phenomenon may help to explain the role of nicotine as a major risk factor for AAA formation and has health implications for ECIGs and other modes of nicotine delivery.

## Introduction

In United States and globally, the use of ECIGs has increased annually across all age groups, and recently ECIGs have become more commonly used among 12th graders than tobacco cigarettes (Arrazola et al., 2015; Bunnell et al., 2015; Gravely et al., 2015; McMillen et al., 2015). One reason for this rising popularity may be the general misconception that ECIGs are a relatively harmless alternative to conventional smoking that lacks toxic tobacco combustion products. In particular, ECIGs are promoted as a “healthy” smoking cessation aid among nicotine-dependent young adult conventional smokers, however recent research suggests a potential to damage DNA at a chromosomal and a gene level in urine (Canistro et al., 2017). In this context, a more complete understanding of the risks of nicotine is mandatory, particularly given that ECIGs can exceed the nicotine delivery profile of tobacco cigarettes (Talih et al., 2015; Dawkins et al., 2016).

In the realm of cardiovascular medicine, a recent study found that ECIG use acutely increases arterial stiffness (Lundbäck et al., 2017). Notably, stiff conduit arteries lose their capability to mechanically buffer against the pulsatile nature of cardiac ejection, resulting in widespread augmentation of hemodynamic stress on end-organs. As such, arterial stiffness has been identified as a strong independent risk factor for many cardiovascular conditions, e.g., heart failure, myocardial infarction, stroke, and AAA formation (Lyle and Raaz, 2017).

The aforementioned acute effects on arterial stiffness appeared to be transient, and due to nicotine’s short-term effects on vascular function. However, the chronic and potentially irreversible progressive effects on arterial stiffness that may result from structural vascular alterations due to long-term nicotine exposure are unknown.

Here, we report that prolonged nicotine exposure in mice induces fragmentation of the elastic layers of the aortic wall, irreversibly increasing structural arterial stiffness in mice. Importantly, we find that nicotine-induced aortic stiffening predominantly affects the abdominal aortic segment—thereby revealing a novel mechanism to explain the role of nicotine as a major risk factor for AAA formation.

## Materials and Methods

The authors declare that all supporting data are available within the article and its online Supplementary Files.

### Animals

C57BL/6 wild-type male mice were purchased from the Jackson Laboratory. Animals were housed in a temperature-controlled and humidity-controlled room under a 12-h light/dark cycle (6:30 am/6:30 pm). All animal protocols were approved by the VA Institutional Animal Care and Use Committee and followed the National Institutes of Health and U.S. Department of Agriculture Guidelines for Care and Use of Animals in Research.

### Osmotic Mini-Pump Implantation

10-week-old male C57BL/6J mice (24–26 g) were anesthetized with inhaled 2% isoflurane and osmotic mini pumps (ALZET, United States) were implanted subcutaneously slightly posterior to the scapulae. Animals were examined after 10 and 40 days. Nicotine (Sigma-Aldrich, United States) was diluted with PBS (Gibco, United States) and the perfusion rate was set to (25 mg/kg/day). The control group was infused with PBS. Effectiveness of nicotine delivery was confirmed by cotinine levels (Calbiotech, United States, CO096D-100).

### SB-3CT Treatment

SB-3CT was purchased from ApexBio Technology, United States and administrated by i.p. injection at day 0 and every other day thereafter at a concentration of 25 mg/kg in 65% polyethylene glycol 200 (Fisher Scientific, United States), 25% dimethyl sulfoxide (Sigma-Aldrich, United States), and 10% ddH_2_O. SB-3CT possesses an inhibitory constant (Ki) of 14 nM for MMP-2 and 600 nM for MMP-9 and is metabolized to an even more potent and competitive gelatinase inhibitor *in vivo.* Off-target effects are unknown and Ki-values for other MMPs are within a micromolar range (206 μM for MMP-1, 15 μM for MMP-3, and 96 μM for MMP-7).

### *In vivo* Ultrasound Studies (PWV)

PWV was examined to globally [including both the TS and AS (Supplementary Figure 1A)] assess aortic stiffness by simultaneous tracking of the R-wave of the ECG and the pulse wave at two specific locations: the LSA and the bif. We determined the PVW as a ratio of the distance (d) and time (t) delay of the pulse wave between both locations. PWV was calculated as PWV = [d(bif)-d(LSA)]/[t(bif)-t(LSA)]. All measurements have been conducted following the two-mean principle.

### *Ex vivo* Pressure Myography

Pressure myography was performed as previously described (Raaz et al., 2015a). In short, TS and AS were explanted from post-treatment male C57BL/6J mice. The midparts of the descending TS and AS were dissected and further processed (Supplementary Figure 1A). TS and AS samples both approximately 0.6–0.8 cm in length were placed on specially designed stainless-steel cannulas and secured with silk surgical suture (10-0). Aortic segments were mounted in the heated vessel chamber of a pressure arteriograph system (Model 100P, Danish Myotechnology, Copenhagen, Denmark) and extended to *in vivo* length. Physiological saline solution at 37°C, aerated with 5% CO_2_/95% O_2_ was used to fill the vessel chamber and for aortic perfusion. Subsequently, aortic segments were pressurized from 0 to 144 mmHg in 18 mmHg increments, and the vessel’s outer diameter was simultaneously tracked by continuous computer video analysis. The strain (S) was calculated as a ratio of outer diameter at baseline (D_b_) to outer diameter at every given pressure level (D_p_) (S = (D_p_−D_b_)/D_b_).

### Immunofluorescence Staining of Aortic Tissue

Aortic cross sections (7 μm) were incubated with rabbit anti-MMP-2 antibody (Abcam, dilution 1:500, ab37150) and rabbit anti-MMP-9 antibody (dilution 1:500; Abcam, ab38898) at 4°C overnight. Goat anti-rabbit IgG secondary antibody (Alexa Fluor 633, Thermo Fisher, dilution 1:1,000, A-21070) was performed at room temperature for 1 h. Counterstaining was performed with Hoechst reagent (Thermo Fisher). Negative controls were performed with the omission of the primary antibody. Imaging was performed using a Zeiss microscope (Oberkochen, Germany). MMP-2/-9 protein expression level was analyzed quantifying red fluorescence intensity in the ROI.

### Elastin Imaging

Elastin layers were visualized using modified Verhoeff–Van Gieson stain (VVG) according to manufacturer’s protocol (Abcam). The number of MLUs was counted in both thoracic and abdominal aortic segments. Elastin fragmentation was quantified in the histological images using elastin morphometric analysis (ImageJ). As previously described (Raaz et al., 2015a), each continuous set of pixels that formed a connected group was defined as an object. An elastin fragmentation index was defined as the ratio of the number of elastin objects to the area of elastin objects. The number of elastin objects was defined by the count of elastin objects within the media ROI. The area of elastin objects was equivalent to the total pixel count across all elastin objects within the media ROI.

### Metalloproteinase *in situ* Zymography

*In situ* zymography was performed according to manufacturer’s instructions. In brief, aortic sections were exposed to DQ gelatin (10 μg/ml; Invitrogen) and 1% agarose solution (ratio 1:10). Representative images were obtained after digestion of fluorescein-labeled gelatin by the endogenous gelatinases MMP-2 and MMP-9. MMP activity was quantified measuring the fluorescence intensity in the ROI.

### RNA Isolation From Aortic Tissue

Animals were anesthetized, and aortic tissue was dissected. Tissue was snap-frozen and homogenized in TRIzol Reagent (Invitrogen, United States). After phase separation, total RNA was washed and eluted in Nuclease-Free Water (Ambion, United States).

### Quantitative Real-Time PCR

To synthesize first-strand cDNA from mRNA, the SuperScript VILO cDNA Synthesis Kit (Invitrogen) was used. TaqMan qRT-PCR assay was used to quantitate mRNA levels. Specific oligonucleotide primers for *MMP-2* (NM_008610.2) and *MMP-9* (NM_013599.3) were obtained from Fisher Scientific, United States. Data are normalized to 18S and all fold changes were calculated using the ΔΔCt method.

### Statistical Analyses

The data are shown as the mean ± SEM. Statistical analysis was performed using GraphPad Prism 6.0 (San Diego, CA, United States). Pressure-strain graphs of experimental groups were analyzed by calculating the AUC. Shapiro–Wilk normality test was applied to examine the normality of the data. One and two-way ANOVA with Holm–Sidak multiple comparison test was applied to compare the study groups after 10 and 40 days, respectively. Student’s *t*-test was used to compare elastin baseline levels for the AS and TS. The level of significance was set to *p* < 0.05.

## Results

### Nicotine Infusion Increases Overall Aortic Stiffness in Mice

**FIGURE 1:**
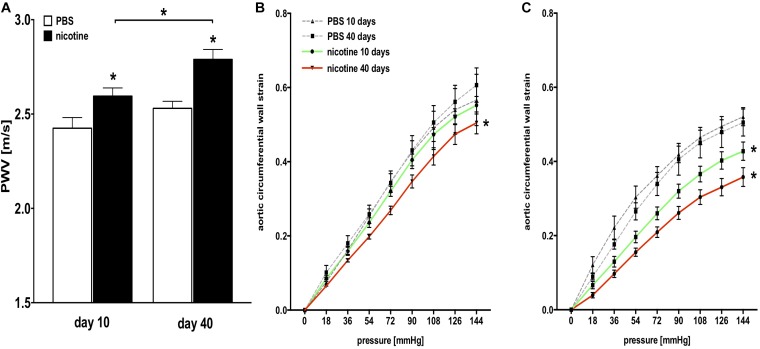
Structural aortic stiffness is increased in nicotine-infused mice. **(A)** Pulse wave velocity (PWV) measurements in nicotine-treated and PBS control mice. (*n* = 4–6 mice/group); ^∗^*p* < 0.05 vs. PBS control or day 10 vs. day 40, respectively; values are mean ± SEM. **(B)** Passive pressure-strain curves derived from pressure myography of the thoracic aortic segment (TS) in nicotine-treated and PBS control mice 10 and 40 days after pump implantation. (*n* = 5–9 mice/group at day 10 and 4–7 mice/group at day 40); ^∗^*p* < 0.05 for area under the curve (AUC) vs. corresponding PBS control at day 10 and day 40, respectively; values are mean ± SEM. **(C)** Passive pressure-strain curves derived from pressure myography of the abdominal aortic segment (AS) in nicotine-treated and PBS control mice 10 and 40 days after pump implantation. (*n* = 5–9 mice/group at day 10 and 4–7 mice/group at day 40); ^∗^*p* < 0.05 for AUC vs. corresponding PBS control at day 10 and day 40, respectively; values are mean ± SEM. **(A–C)** Two-way ANOVA with Holm–Sidak’s multiple comparison test.

We measured aortic PWV (from the LSA to the aortic bifurcation; Supplementary Figure 1A) as the clinical gold standard to globally assess aortic stiffness via ultrasound after 10 and 40 days of nicotine infusion via osmotic pump. As expected, we found significantly elevated serum cotinine levels in nicotine-infused mice after 10 and 40 days when compared to PBS-infused controls, indicating effective nicotine delivery (Supplementary Figure 1B). Mice that received nicotine infusion showed significantly elevated overall aortic stiffness (i.e., increased PWV) both 10 and 40 days after osmotic pump implantation compared to mice that received PBS infusion (Figure 1A). Additionally, aortic stiffness increased significantly from day 10 to day 40 (Figure 1A).

### Nicotine Infusion Induces Segmental Aortic Stiffness in Mice

We directly quantified the passive, structural stiffness of the aortic wall by performing aortic *ex vivo* mechanical testing using pressure myography. We verified that mice responded to nicotine infusion over time with increased aortic stiffness, both in the TS (Figure 1B) and AS (Figure 1C), as stiffness-related AUC decreased with nicotine infusion.

Given the distinct embryologic and structural differences between the thoracic and abdominal aorta, we next investigated whether nicotine exposure differentially affects those aortic segments. As such, we compared *ex vivo* mechanical stiffness of both segments under nicotine and control conditions.

**FIGURE 2:**
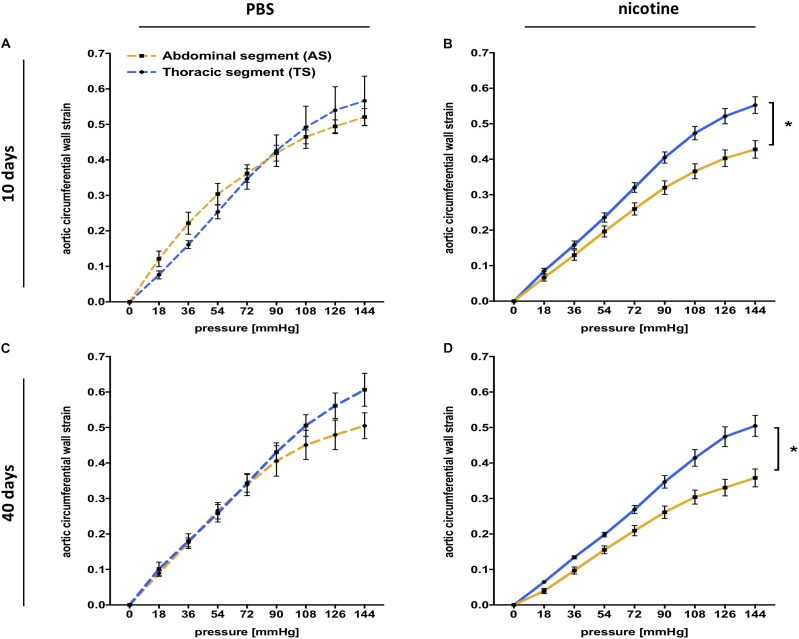
Segmental aortic stiffness is increased after nicotine infusion. **(A)** Pressure myography indicates similar global stiffness between the thoracic (TS) and the abdominal (AS) aortic segment for PBS control mice 10 days after pump implantation [equal area under the curve (AUC)]. (*n* = 5 mice/group); values are mean ± SEM. **(B)** Pressure myography indicates a significant stiffness gradient (difference for AUC) between the TS and the AS in nicotine-treated mice 10 days after pump implantation (*n* = 9 mice/group); ^∗^*p* < 0.05 AS vs. TS; values are mean ± SEM. **(C)** Pressure myography indicates similar global stiffness between the TS and the AS for PBS control mice 40 days after pump implantation. (*n* = 4–5 mice/group) (equal AUC); values are mean ± SEM. **(D)** Pressure myography indicates a significant stiffness gradient (difference for AUC) between the TS and the AS for nicotine-treaded mice 40 days after pump implantation. (*n* = 6–7 mice/group); ^∗^*p* < 0.05; values are mean ± SEM. **(A–D)** Two-way ANOVA with Holm–Sidak’s multiple comparison test.

Control mice receiving PBS infusion did not exhibit significant differences in aortic stiffness between the TS and AS, either at day 10 or at day 40 (Figures 2A,C), indicating homogenous aortic stiffness levels. In contrast, mice that received nicotine infusion developed disproportionately increased global aortic stiffness in the AS compared to the TS at 10 and 40 days after pump implantation (as indicated by increased AUC differential in the AS) and resulting in a stiffness gradient along the aorta (Figures 2B,D).

### Elastin Architecture Is Disrupted in Nicotine-Treated Mice

**FIGURE 3:**
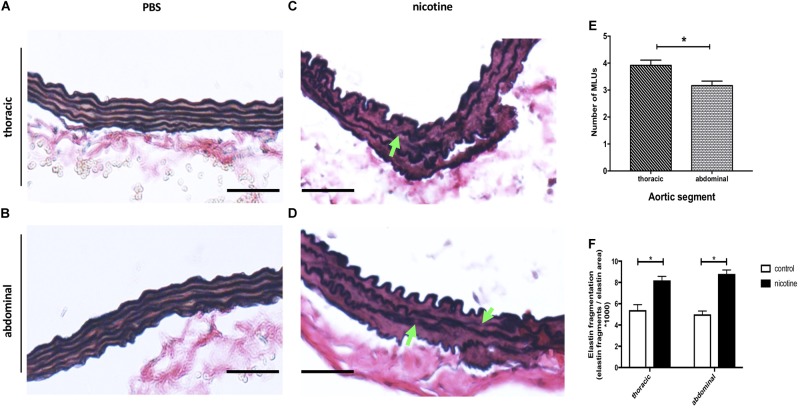
Nicotine infusion induces aortic elastin fragmentation. **(A–D)** Representative images of thoracic **(A,C)** and abdominal **(B,D)** aortic sections taken from mice treated with PBS **(A,B)** or nicotine **(C,D)** and stained with modified Elastin Verhoeff’s Van Gieson (VVG). Green arrows indicate locations of elastin fragmentation. Original magnification 200×, scale bar 100 μm. **(E)** Quantification of medial lamellar units (MLUs) of thoracic and abdominal aortic segments from PBS-treated control mice 40 days after pump implantation (the mean was calculated from 4 high-power fields/aorta, *n* = 3 different aortas per group). ^∗^*p* < 0.05 AS vs. TS, values are mean ± SEM; Student’s *t*-test. **(F)** Elastin fragmentation index 40 days after pump implantation (the mean was calculated from 3 high-power fields/aorta, *n* = 5 different aortas per group). ^∗^*p* < 0.05 vs. respective PBS control; values are mean ± SEM. One-way ANOVA with Holm–Sidak’s multiple comparison test.

To investigate the mechanisms leading to globally increased aortic stiffness as well as stiffness segmentation in nicotine-treated mice, we examined the aortic elastin structure. Elastin is a major component of the aortic wall, critically defining the passive mechanical properties (elasticity) of the vessel. We modified VVG staining (Figures 3A–D), and quantified elastin organization and damage as described previously (Raaz et al., 2015a).

Under basal (PBS) conditions we found well preserved elastin architecture in the thoracic and abdominal aortic media (Figures 3A,B). However, the number of elastin lamellae was higher in the TS vs. AS (Figures 3A,B,E).

Following 40 days of nicotine exposure, we detected significant elastin damage (thinning and fragmentation) in both the thoracic as well as the abdominal aorta (Figures 3C,D,F). However, there was no significant difference in the ratio of elastin fragmentation between the TS and AS.

### Nicotine Treatment Increases Aortic MMP-2 and MMP-9 Expression and Elastolytic Activity

MMPs are enzymes involved in turnover and degradation of most extracellular matrix (ECM) proteins, including elastin. We therefore sought to further delineate their potential role in the development of nicotine-induced aortic stiffness.

**FIGURE 4:**
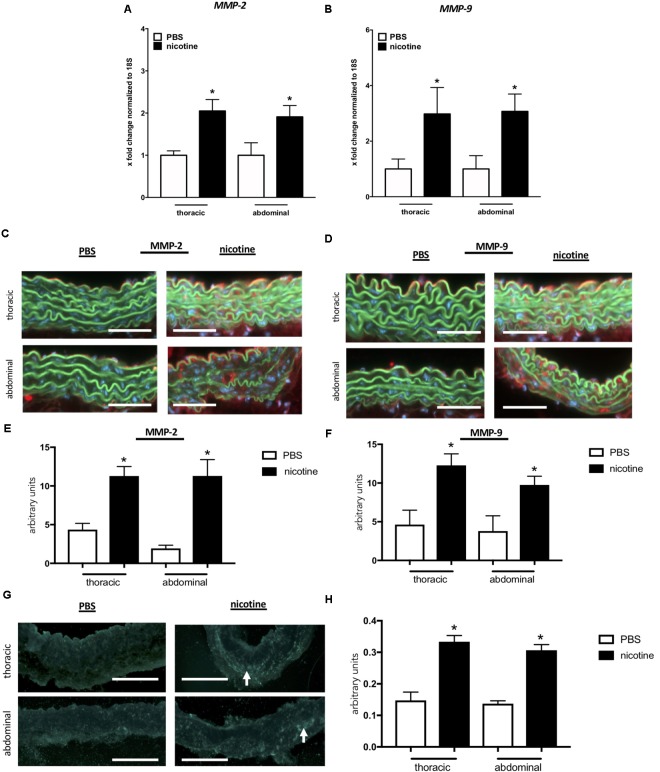
Nicotine treatment induces MMP-2 and MMP-9 gene and protein expression resulting in increased MMP activity. **(A,B)** mRNA expression levels of MMP-2 and MMP-9 are upregulated in the thoracic (TS) and the abdominal aortic segment (AS) after nicotine treatment. (*n* = 5 mice/group); ^∗^*p* < 0.05 vs. respective PBS control; values are mean ± SEM. **(C,D)** Representative images of aortic sections stained for MMP-2 **(C)** and MMP-9 **(D)**. Strong red fluorescence (MMP-2/-9) indicates protein up-regulation in nicotine-treated mice aortae. Original magnification 200×, scale bar 100 μm. **(E,F)** Quantification of MMP-2 **(E)** and MMP-9 **(F)** protein expression level in the aortic wall based on red fluorescence in sections taken from AS and TS of nicotine-treated and PBS control mice 40 days after pump implantation (the mean was calculated from 3 high-power fields/aorta, *n* = 5 different aortas per group). ^∗^*p* < 0.05 vs. respective PBS control; values are mean ± SEM. **(G)** Representative images of aortic zymography of TS and the AS taken from nicotine-treated or PBS control mice. Green–blue fluorescence indicates regions of active MMPs (white arrows). Original magnification 200×, scale bar 100 μm. **(H)** Quantification of MMP activity based on green–blue fluorescence in zymography images taken from AS and TS of nicotine-treated and PBS control mice 40 days after pump implantation (the mean was calculated from 3 high-power fields/aorta, *n* = 5 different aortas per group). ^∗^*p* < 0.05 vs. respective PBS control; values are mean ± SEM. **(A,B,E,F,H)** One-way ANOVA with Holm–Sidak’s multiple comparison test.

First, we analyzed *MMP-2* and *MMP-9* expression via qRT-PCR and found that both genes were significantly upregulated in both the TS and AS at 40 days after nicotine pump implantation when compared to PBS controls (Figures 4A,B). On the protein level, this translated into marked upregulation of MMP-2 and MMP-9 protein expression as detected by increased red immunofluorescence in the media of aortic sections (Figures 4C–F), again without particular differences between TS and AS.

In order to assess the functional impact of nicotine-triggered MMP induction, we performed *in situ* zymography in aortic sections. Aortic sections from nicotine-treated mice exhibited significantly higher elastolytic enzyme activity than PBS controls indicated by enhanced green–blue fluorescence (Figures 4G,H). Again, we detected no significant differences between the thoracic and abdominal aortic segments regarding this endpoint.

### MMP-2/-9 Inhibition via SB-3CT Prevents Nicotine-Induced Aortic Stiffening

**FIGURE 5:**
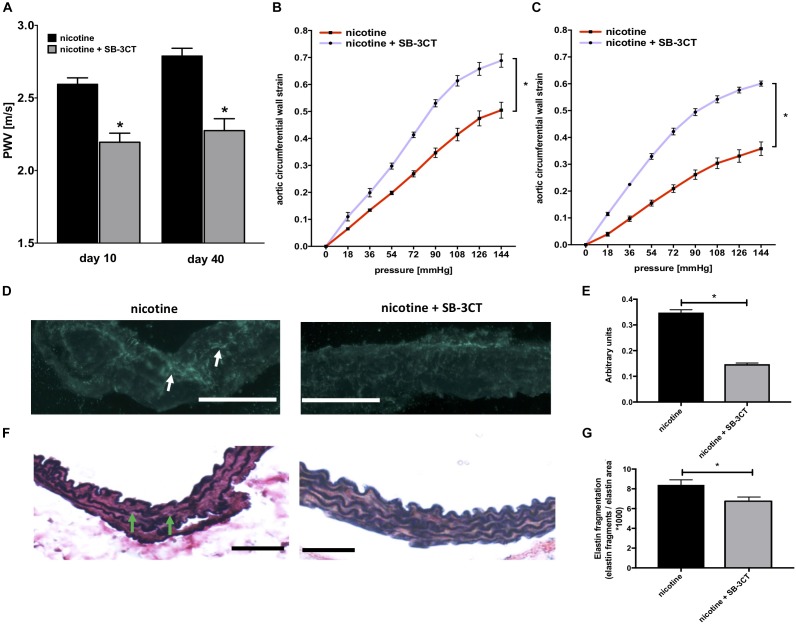
MMP inhibition via SB-3CT prevents aortic wall remodeling and stiffening in nicotine-treated mice. **(A)** PWV for nicotine-treated mice ± additional SB-3CT administration (*n* = 4–8 mice/group); ^∗^*p* < 0.05 vs. nicotine; values are mean ± SEM. **(B,C)** Pressure-strain curves for the thoracic (TS) **(B)** and the abdominal **(C)** aortic segment (AS) from nicotine-infused mice ± SB-3CT administration 40 days after pump implantation (4–7 mice/group); ^∗^*p* < 0.05 area under the curve (AUC) nicotine vs. AUC nicotine + SB-3CT; values are mean ± SEM. **(D)** Representative images of aortic zymography of the TS taken from nicotine-treated ± SB-3CT administrated mice. Green–blue fluorescence indicates regions of active MMPs (white arrows). Original magnification 200×, scale bar 100 μm. **(E)** Quantification of MMP activity based on green–blue fluorescence intensity in zymography images taken from nicotine-treated ± SB-3CT administrated mice 40 days after pump implantation (the mean was calculated from 3 high-power fields/aorta, *n* = 5 different aortas per group); ^∗^*p* < 0.05 nicotine vs. nicotine + SB-3CT; values are mean ± SEM. **(F)** Representative images of thoracic aortic sections from nicotine ± SB-3CT-treated mice stained with Elastica van-Giesson (EvG). Green arrows indicate locations of elastin fragmentation. Original magnification 200×, scale bar 100 μm. **(G)** Elastin fragmentation index (the mean was calculated from 3 high-power fields/aorta, *n* = 5 different aortas per group). ^∗^*p* < 0.05 nicotine vs. nicotine + SB-3CT; values are mean ± SEM. **(E,G)** One-way ANOVA with Holm–Sidak’s multiple comparison test.

In a prophylactic approach, we next investigated whether MMP-2/-9 inhibition via SB-3CT was sufficient to prevent nicotine-induced aortic stiffness and stiffness segmentation. As before, we used PWV (Figure 5A) and *ex vivo* myograph measurements (Figures 5B,C) to quantify aortic stiffness 40 days after pump implantation and found that SB-3CT was sufficient to reduce overall and segmental aortic stiffness in nicotine-treated mice.

### SB-3CT Ameliorates Nicotine-Induced Elastin Destruction and MMP Activity

Mechanistically, *in situ* zymography confirmed markedly reduced MMP activity in aortic sections taken from nicotine-treated mice with additional SB-3CT administration after 40 days of nicotine exposure. This was indicated by a significantly reduced green/blue fluorescence in aortic sections (Figures 5D,E). On a structural level, this translated into preserved elastin architecture as detected via modified VVG staining (Figures 5F,G).

## Discussion

This study provides evidence that chronic nicotine exposure, at concentrations similar to those experienced by ECIG users, promotes aortic stiffening, with enhanced impact on the abdominal aortic segment with regards to the time of onset and quantity. Mechanistically, nicotine infusion led to induction of MMP expression and activity, with associated elastin degradation in the aortic wall, reduction in elastic properties, and stiffening of the aortic segments. This process was effectively prevented with the administration of SB-3CT, a potent and competitive MMP-2/-9 inhibitor.

Nicotine has long been known to induce significant cardiovascular effects, including the release of catecholamines resulting in acute increase of blood pressure and heart rate, although long-term effects seem to be contrary (Leone, 2011; Morris et al., 2015). ECIGs and conventional tobacco cigarettes generate similar plasma levels of the nicotine metabolization product cotinine in humans (Flouris et al., 2013). This supports findings from Barua et al. who found plasma cotinine levels of 140 ng/ml in light and 286 ng/ml in heavy smokers, while a previous literature review suggested peak plasma cotinine levels of approximately 300 ng/ml for ECIG users after a number of free puffs (Barua et al., 2002; Marsot and Simon, 2016). Notably, this study reveals similar plasma cotinine levels for nicotine-treated mice suggesting valid transferability. Recently, it was reported that ECIG usage acutely increases arterial stiffness in healthy volunteers (Lundbäck et al., 2017). This finding leads to fundamental questions regarding the safety of ECIG usage—taking into consideration that arterial stiffness is a risk factor for a wide set of cardiovascular diseases (Lyle and Raaz, 2017). In the acute setting, arterial stiffness may increase passively as a function of rising blood pressure and then similarly normalize. In contrast, chronic arterial stiffness may arise out of (irreversibly) altered biomechanical properties of the arterial wall, resulting from sustained structural remodeling processes (Wolinsky and Glagov, 1964; Wagenseil and Mecham, 2009). In this regard, our ultrasound and pressure myography data, along with histochemical evidence, clearly indicate that nicotine exposure induces chronic structural stiffening of the aortic wall.

Elastin and collagen represent the major structural components of the arterial ECM that critically define vessel biomechanical characteristics. Elastic fibers are found in the medial layer of large elastic arteries, primarily the aorta. The fibers form a network in which each layer of elastic fibers is circumferentially surrounded by smooth muscle cells (SMCs) and collagen fibers forming a lamellar unit (Wagenseil and Mecham, 2012). As elastin fibers straighten with increasing pressure they become load-bearing (Clark and Glagov, 1985). This linear distension is limited by collagen fibers, protecting the vessel from rupture when the pressure exceeds normal physiological levels (Armentano et al., 1991). Within the standard physiologic blood pressure range, medial elastin degradation and fragmentation (mainly caused by enhanced MMP activity) may be the major factors behind early aortic stiffening (Murphy et al., 1991; Ishii and Asuwa, 1996; Lyle and Raaz, 2017). In this regard, we find that nicotine treatment induces significant damage to the aortic wall elastin network, resulting in increased structural arterial stiffness. Unfortunately, elastic fibers are almost exclusively assembled during tissue development, and then must function for the entire lifespan of the organism with low potential for repair (Wagenseil and Mecham, 2009). As such, elastin damage inflicted by chronic nicotine usage may be permanent—even after removal of the noxious trigger.

There are a number of MMPs with known elastolytic activity, including some with important roles in vascular remodeling (Perlstein and Lee, 2006; Jacob-Ferreira et al., 2010) MMP-2 (gelatinase A) is constitutively expressed by SMCs. MMP-9 (gelatinase B) can be produced by macrophages, fibroblasts, or SMCs with a secretory phenotype. Interestingly, high serum levels of MMP-2 and MMP-9 have been associated with increased arterial stiffness in healthy individuals, and patients with isolated systolic hypertension (Yasmin et al., 2005). Wang et al. (2012) found that AMP-activated protein kinase α2 showed increased nuclear co-localization with nicotine, upregulating MMP expression, and instigating the formation of AAA in genetically modified mice. Our data are in line with these findings, as they show upregulated aortic expression and activity of MMP-2 and MMP-9 with prolonged nicotine exposure, and are also consistent with other reports showing similar findings in vascular cells, such as endothelial cells and SMCs (Carty et al., 1996; Jacob-Ferreira et al., 2010; Dom et al., 2011).

In order to test the functional relevance of increased MMP activity as a mechanism promoting nicotine’s effects on arterial stiffness we employed SB-3CT, a pharmacologic MMP-2/-9 inhibitor. Indeed, SB-3CT effectively limited MMP activation and arterial stiffening in nicotine-treated mice, in fact exceeding PBS-control levels for stiffness-related parameters. Given its capacity for inhibition of other MMPs beyond MMP-2/-9, albeit less potently, the authors propose that changes in ECM composition through prolonged inhibition of multiple MMPs might affect aortic wall stiffness through mechanisms beyond elastin fragmentation. In line with this, collagen content and cross-linking or calcification have been identified as substantial sources for arterial stiffness, although they were not investigated in this study (Lyle and Raaz, 2017). Conclusively, results of this study might encourage investigators to explore the involvement of SB-3CT in these processes to decrypt its mechanism of action in more detail.

As another intriguing finding, nicotine exposure did not affect aortic stiffness uniformly. Instead, we showed that nicotine elicits more stiffening of the abdominal aortic segment. While our data demonstrate that the native aorta does not exhibit significant stiffness gradients, nicotine exposure induced aortic stiffness segmentation with a marked gradient between the thoracic and the abdominal aorta (Figure 2). At first glance, this was somewhat surprising, as we did not detect differential MMP-2/-9 expression or activity between the aortic segments in nicotine-treated mice. However, due to differences in embryologic development the aorta exhibits decreasing elastin content from the thoracic to the abdominal segments (Wolinsky and Glagov, 1969; Halloran et al., 1995). Accordingly, we found a higher number of elastin lamellae (or lamellar units) in the thoracic aorta compared to the abdominal segment, which is in agreement with previous reports (Goergen et al., 2010; Collins et al., 2011). Thus, although nicotine uniformly upregulates MMP expression in the aorta, the lower basal elastin content of the abdominal aorta may render this segment particularly susceptible to stiffening effects.

This novel observation may have major (patho)mechanistic implications, particularly for AAA disease. AAA is largely a phenomenon of the aging aorta. Aortic aging is accompanied by progressive elastin fragmentation and subsequent stiffening (O’Rourke, 2007). Previously, we and others have noted that aortic aging (in part due to decreased elastin) induces segmental stiffening of the abdominal aorta (Hickson et al., 2010; Raaz et al., 2015b; Zhang et al., 2016). We found that stiffness gradients along the aorta (resulting from stiffness segmentation) locally enhance aortic mechanical wall stress and are critical drivers of early AAA growth (Raaz et al., 2015b). This study now adds the important insight that nicotine intake may dramatically accelerate the biomechanical alterations linked to aortic aging, setting the stage for later AAA formation. This may explain in part why (a) smoking is a major risk factor for AAA and (b) nicotine, despite its systemic impact, preferentially elicits/augments aneurysms in the abdominal aortic segment. Moreover, our study strengthens the notion that in addition to other toxic tobacco combustion products, nicotine alone may convey risk for AAA formation. This is consistent with our previous findings showing that nicotine exposure augments AAA formation in mouse models (Maegdefessel et al., 2012).

This study has various limitations. First, the nicotine delivery through osmotic mini-pumps in mice does not necessarily mimic nicotine uptake through ECIGs and metabolism in humans, which might limit the translatability of our results. Second, we used the AUC of pressure-strain curves as a global assessment of arterial stiffness for *ex vivo* myograph measurements. Even though correlation between the AUC and arterial stiffness appears likely, this analytic approach might limit our conclusions and somewhat compromises comparisons to results from literature using different stiffness parameters.

In summary, our research indicates that nicotine intake may bring about substantial structural damage to large conduit vessels (such as the aorta), thereby accelerating two major detrimental features of arterial aging: increased arterial stiffness and biomechanical susceptibility for AAA formation. Our data further corroborate the role of nicotine as a significant cardiovascular risk factor and suggest that substantial caution is warranted regarding the use of ECIGs and other forms of nicotine replacement.

## Ethics Statement

This study was carried out in accordance with the recommendations of the R & D Committee of the VA Palo Alto Health Care System. The protocol was approved by the R & D Committee of the VA Palo Alto Health Care System.

## Author Contributions

MW, JS, and PT designed the research study. MW, IS, SG, TY, KT, AD, AP, JM, PM, and YK conducted the experiments. MW, IS, UR, JM, ME, WI, AR, GH, AS, MA, HS, JS, and PT acquired and analyzed the data. MW, IS, UR, JS, TY, KT, YK, AD, SG, AP, JM, GH, AS, AR, MA, HS, and PT revised the manuscript for important intellectual content. MW, IS, UR, JS, WI, ME, PM, and PT assisted in writing the article. All authors approved the final version of the manuscript and agree to be accountable for all aspects of the work.

## Conflict of Interest Statement

The authors declare that the research was conducted in the absence of any commercial or financial relationships that could be construed as a potential conflict of interest. The reviewer CH and handling Editor declared their shared affiliation at the time of the review.

## Abbreviations

AAA: abdominal aortic aneurysms
AS: abdominal aortic segment
AUC: area-under-curve
bif: aortic bifurcation
ddH_2_O: double-distilled water
DNA: complementary deoxyribonucleic acid
ECG: electrocardiogram
ECIGs: e-cigarettes
ECM: extracellular matrix
LSA: left subclavian artery
MLUs: medial lamellar units
MMP: matrix-metalloproteinase
MMP-2: matrix-metalloproteinase-2
MMP-9: matrix-metalloproteinase-9
mRNA: messenger ribonucleic acid
PBS: phosphate buffered saline
PWV: pulse wave velocity
ROI: region of interest
RNA: ribonucleic acid
SB-3CT: SB-3CT (2-[[(4-Phenoxyphenyl)sulfonyl]methyl]thiirane)
TS: thoracic aortic segment
VVG: Verhoeff–Van Gieson.
