# Supplementary figures and images for: Chronic Nicotine Exposure Induces Murine Aortic Remodeling and Stiffness Segmentation—Implications for Abdominal Aortic Aneurysm Susceptibility
Source: Front Physiol. 2018 Oct 31;9:1459. doi: 10.3389/fphys.2018.01459 (PMC6220086; doi:10.3389/fphys.2018.01459)

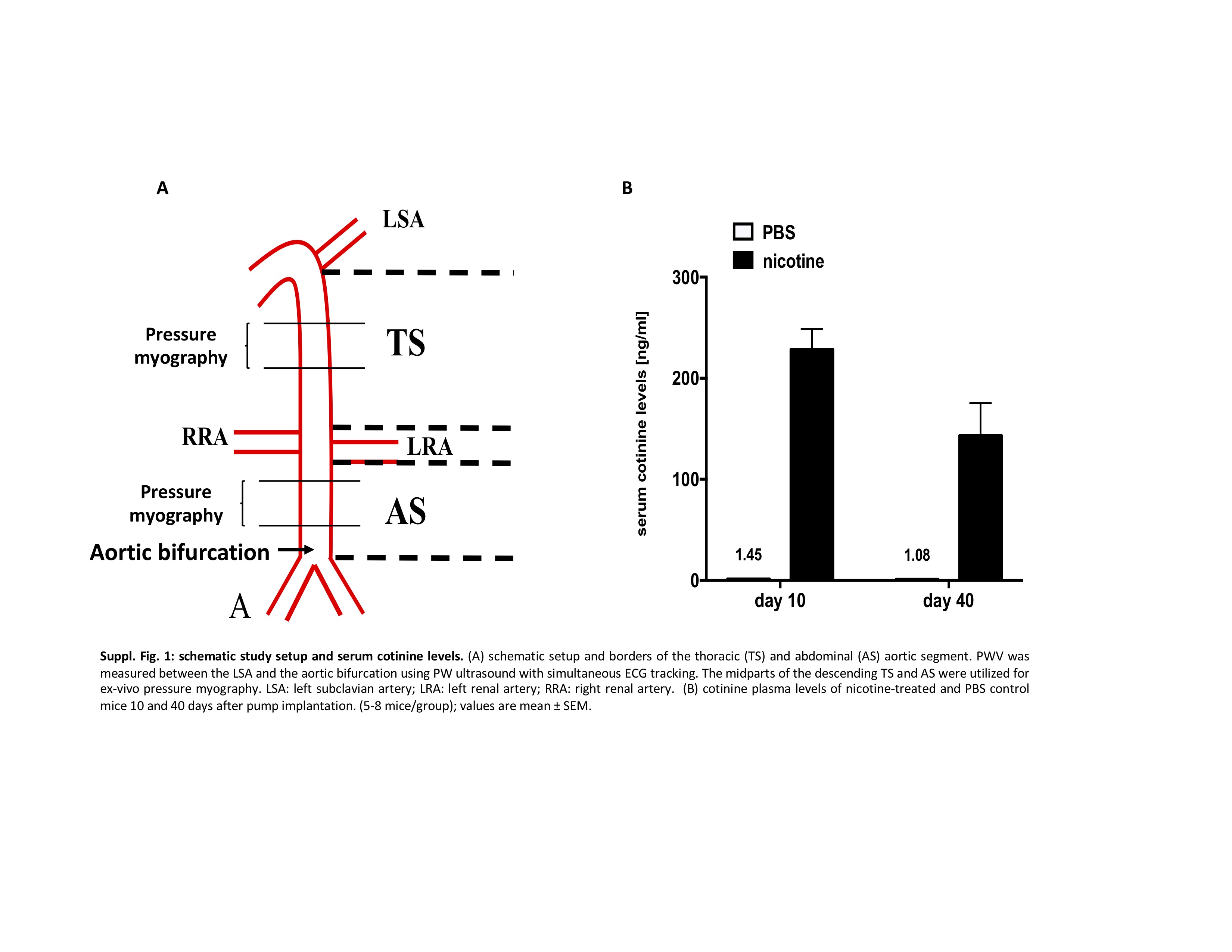

Supplement: Supplementary file 1 [file Image_1.JPEG]
